# Supplementary material for: Vaccination impairs de novo immune response to omicron breakthrough infection, a precondition for the original antigenic sin
Source: Nat Commun. 2024 Apr 10;15:3102. doi: 10.1038/s41467-024-47451-w (PMC11006949; doi:10.1038/s41467-024-47451-w)
Supplement: Supplementary file 1 — Supplementary Information [file 41467_2024_47451_MOESM1_ESM.pdf]

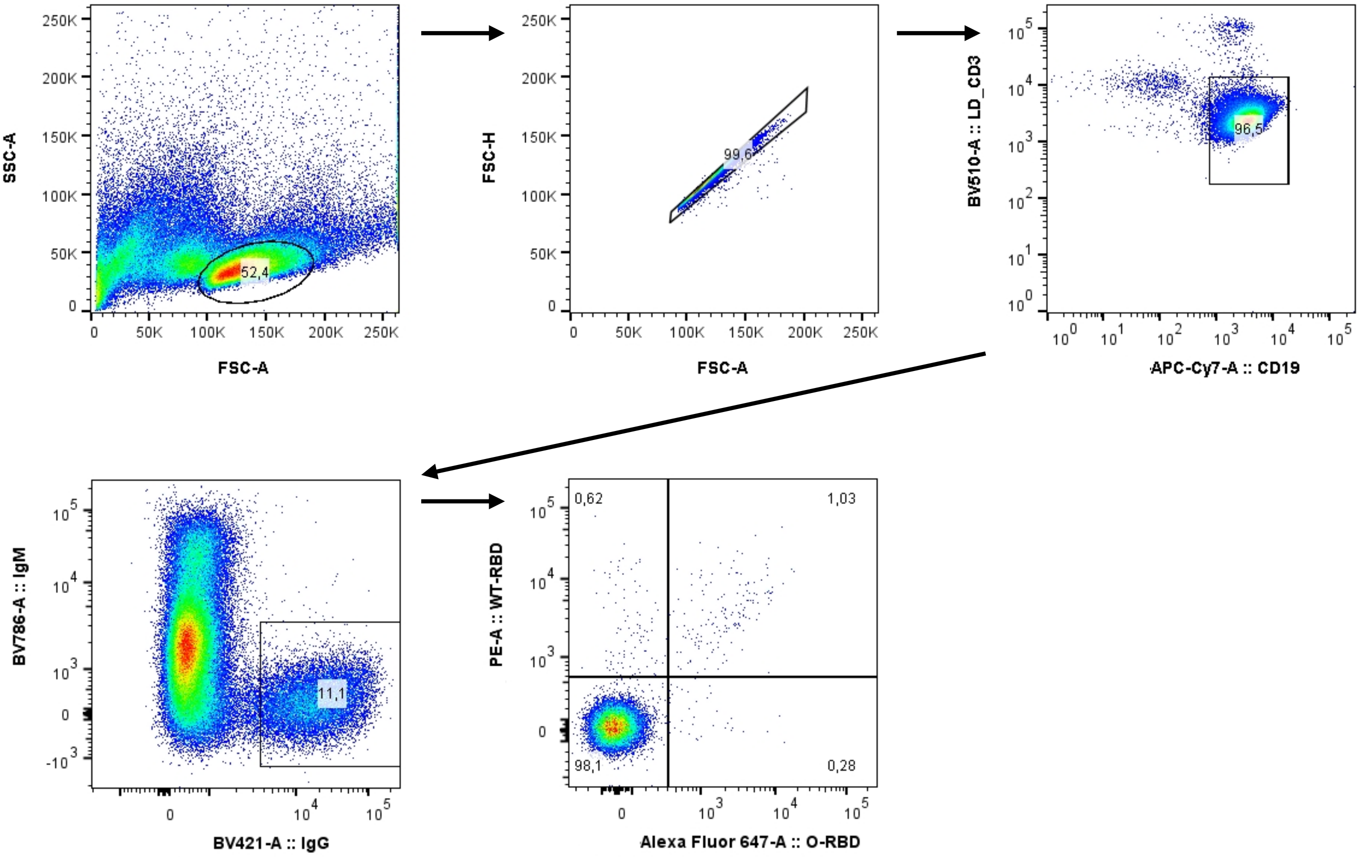

Supplemental Figure 1) Shown is the gating strategy for the identification of IgG+ B cells specific for omicron and/or wild-type SARS-CoV-2 RBD domain of the spike protein. Plots are shown as pseudocolors. Arrows indicate the sequential order in which gates were applied. Numbers within the gates are percentages of the parent population. The above gating strategy was used to derive data presented in Figure 3.

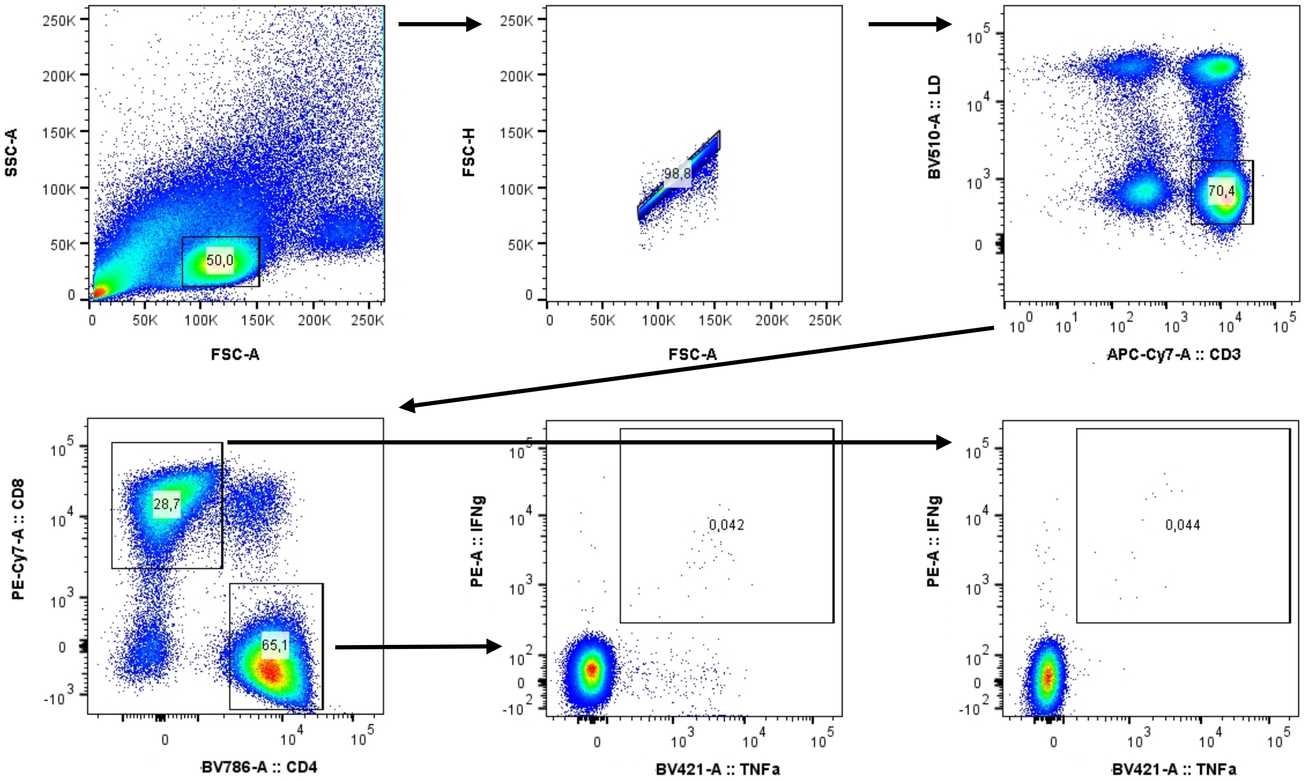

Supplemental Figure 2) Shown is the gating strategy for the identification of CD4 and CD8 T cells specific for conserved or mutated epitopes of the omicron SARS-CoV-2 spike protein. Plots are shown as pseudocolors. Arrows indicate the sequential order in which gates were applied. Numbers within the gates are percentages of the parent population. The above gating strategy was used to derive data presented in Figure 4.

Patient	Group	Sex	Age	1. vaccination	Vaccine	2. vaccination	Vaccine	3. vaccination	Vaccine	Positive PCR	Omicron subvariant	Sample collection
**1**	O-Inf+Vacc	female	51-60	Mar-21	Spikevax	Jun-21	Spikevax	Dec-21	Spikevax	Feb-22	BA.1	May-22
**2**	O-Inf+Vacc	female	41-50	Jan-21	Spikevax	Feb-21	Spikevax	Nov-21	Spikevax	Mar-22	BA.2	May-22
**3**	O-Inf+Vacc	female	61-70	Feb-21	Spikevax	Mar-21	Spikevax	Nov-21	Spikevax	Mar-22	BA.2	May-22
**4**	O-Inf+Vacc	male	71-80	Mar-21	Spikevax	Jun-21	Spikevax	Nov-21	Spikevax	Apr-22	BA.2	May-22
**5**	O-Inf+Vacc	male	51-60	Jan-21	Spikevax	Feb-21	Spikevax	Oct-21	Spikevax	Mar-22	BA.2	May-22
**6**	O-Inf+Vacc	female	41-50	Feb-21	Spikevax	Mar-21	Spikevax	Nov-21	Spikevax	Apr-22	BA.2	May-22
**7**	O-Inf+Vacc	female	61-70	Feb-21	Spikevax	Mar-21	Spikevax	Dec-21	Spikevax	Mar-22	BA.2	May-22
**8**	O-Inf+Vacc	female	11-20	Jan-21	Spikevax	Feb-21	Spikevax	Dec-21	Spikevax	Feb-22	BA.1	May-22
**9**	O-Inf+Vacc	female	41-50	Apr-21	Spikevax	Jun-21	Spikevax	Dec-21	Spikevax	Feb-22	BA.1	May-22
**10**	O-Inf+Vacc	male	31-40	Feb-21	Spikevax	Mar-21	Spikevax	Dec-21	Spikevax	Mar-22	BA.2	Jun-22
**11**	O-Inf+Vacc	male	21-30	Jan-21	Spikevax	Feb-21	Spikevax	Nov-21	Spikevax	Feb-22	BA.1	May-22
**12**	O-Inf+Vacc	female	21-30	Mar-21	Comirnaty	May-21	Comirnaty	Dec-21	Comirnaty	Feb-22	BA.1	May-22
**13**	O-Inf+Vacc	female	31-40	Mar-21	Spikevax	Apr-21	Spikevax	Nov-21	Spikevax	Dec-22	BA.5/BQ.1	Jan-23
**14**	O-Inf+Vacc	female	21-30	Feb-21	Spikevax	Mar-21	Spikevax	Dec-21	Comirnaty	Nov-22	BA.2	Jan-23
**15**	O-Inf+Vacc	male	61-70	Jan-21	Spikevax	Feb-21	Spikevax	Apr-22	Spikevax	Dec-22	BA.5/BQ.1	Jan-23
**16**	O-Inf+Vacc	male	41-50	Jan-21	Spikevax	Feb-21	Spikevax	May-22	Spikevax	Dec-22	BA.5/BQ.1	Jan-23
**17**	O-Inf+Vacc	female	51-60	Mar-21	Spikevax	Jun-21	Spikevax	Dec-21	Spikevax	Dec-22	BA.5/BQ.1	Jan-23
**18**	O-Inf+Vacc	female	21-30	Apr-21	Spikevax	May-21	Spikevax	Mar-22	Spikevax	Dec-22	BA.5/BQ.1	Jan-23
**19**	O-Inf+Vacc	female	21-30	Feb-21	Spikevax	Mar-21	Spikevax	Nov-21	Spikevax	Nov-22	BA.2	Jan-23
**20**	O-Inf+Vacc	female	21-30	Jan-21	Spikevax	Feb-21	Spikevax	Oct-21	Spikevax	Nov-22	BA.2	Jan-23
**21**	O-Inf+Vacc	male	31-40	Feb-21	Spikevax	Mar-21	Spikevax	Oct-21	Spikevax	Nov-22	BA.2	Jan-23
**22**	O-Inf+Vacc	male	21-30	Feb-21	Spikevax	Mar-21	Spikevax	Oct-21	Spikevax	Jan-23	BA.5/BQ.1	Jan-23
**23**	O-Inf+Vacc	female	31-40	Feb-21	Spikevax	May-21	Spikevax	Oct-21	Spikevax	Jan-23	BA.5/BQ.1	Jan-23
**24**	O-Inf+Vacc	male	31-40	Feb-21	Comirnaty	Mar-21	Comirnaty	Oct-21	Spikevax	Dec-22	BA.5/BQ.1	Jan-23
**25**	O-Inf+Vacc	female	21-30	Feb-21	Spikevax	Mar-21	Spikevax	Oct-21	Spikevax	Dec-22	BA.5/BQ.1	Jan-23
**26**	O-Inf+Vacc	female	21-30	Feb-21	Comirnaty	Mar-21	Comirnaty	Oct-21	Comirnaty	Oct-22	BA.2	Jan-23
**27**	O-Inf+Vacc	female	21-30	Mar-21	Spikevax	Apr-21	Spikevax	Oct-21	Comirnaty	Oct-22	BA.2	Jan-23
**28**	O-Inf+Vacc	male	21-30	Apr-21	Spikevax	May-21	Spikevax	Dec-21	Spikevax	Nov-22	BA.2	Jan-23
**29**	O-Inf+Vacc	female	21-30	Mar-21	Spikevax	Aug-21	Spikevax	Feb-22	Spikevax	Nov-22	BA.2	Jan-23
**30**	O-Inf+Vacc	female	11-20	Apr-21	Spikevax	Oct-21	Spikevax	Mar-22	Spikevax	Nov-22	BA.2	Jan-23
**31**	O-Inf+Vacc	female	61-70	Mar-21	Spikevax	Jul-21	Spikevax	Mar-22	Spikevax	Dec-22	BA.5/BQ.1	Jan-23
**32**	O-Inf+Vacc	female	31-40	Jan-21	Spikevax	Feb-21	Spikevax	Nov-21	Spikevax	Jan-22	BA.1*	Mar-22
**33**	O-Inf+Vacc	female	31-40	Jan-21	Spikevax	Feb-21	Spikevax	Nov-21	Spikevax	Jan-22	BA.1*	Mar-22
**34**	O-Inf+Vacc	male	41-50	Feb-21	Spikevax	Mar-21	Spikevax	Nov-21	Spikevax	Jan-22	BA.1*	Feb-22
**35**	O-Inf+Vacc	female	31-40	Feb-21	Spikevax	Mar-21	Spikevax	Nov-21	Spikevax	Jan-22	BA.1*	Feb-22
**36**	O-Inf+Vacc	male	31-40	Jan-21	Spikevax	Mar-21	Spikevax	Nov-21	Spikevax	Jan-22	BA.1*	Feb-22
**37**	O-Inf+Vacc	male	31-40	Feb-21	Spikevax	Mar-21	Spikevax	Nov-21	Spikevax	Dec-21	BA.1*	Feb-22
**1**	Vacc	female	61-70	Jan-21	Spikevax	Feb-21	Spikevax	Nov-21	Spikevax	/	/	May-22
**2**	Vacc	male	61-70	Jan-21	Spikevax	Feb-21	Spikevax	Nov-21	Spikevax	/	/	May-22
**3**	Vacc	female	41-50	Mar-21	Spikevax	Jun-21	Spikevax	Nov-21	Comirnaty	/	/	May-22
**4**	Vacc	male	51-60	Feb-21	Spikevax	Mar-21	Spikevax	Oct-21	Spikevax	/	/	Nov-21
**5**	Vacc	female	31-40	Feb-21	Spikevax	Mar-21	Spikevax	Oct-21	Spikevax	/	/	Nov-21
**6**	Vacc	male	21-30	Feb-21	Spikevax	Mar-21	Spikevax	Oct-21	Spikevax	/	/	Nov-21
**7**	Vacc	female	21-30	Feb-21	Spikevax	Mar-21	Spikevax	Oct-21	Spikevax	/	/	Nov-21
**8**	Vacc	male	51-60	Jan-21	Spikevax	Feb-21	Spikevax	Oct-21	Spikevax	/	/	Nov-21
**9**	Vacc	female	41-50	Feb-21	Spikevax	Feb-21	Spikevax	Oct-21	Spikevax	/	/	Nov-21
**10**	Vacc	female	31-40	Feb-21	Spikevax	Mar-21	Spikevax	Oct-21	Spikevax	/	/	Nov-21
**11**	Vacc	male	41-50	Jan-21	Spikevax	Feb-21	Spikevax	Oct-21	Spikevax	/	/	Nov-21
**12**	Vacc	female	51-60	Jan-21	Spikevax	Feb-21	Spikevax	Nov-21	Spikevax	/	/	Dec-21
**13**	Vacc	female	21-30	Feb-21	Spikevax	Mar-21	Spikevax	Nov-21	Comirnaty	/	/	Dec-21
**14**	Vacc	male	41-50	Jan-21	Spikevax	Feb-21	Spikevax	Nov-21	Spikevax	/	/	Dec-21
**15**	Vacc	female	51-60	Jan-21	Spikevax	Feb-21	Spikevax	Nov-21	Spikevax	/	/	Dec-21
**16**	Vacc	male	41-50	Jan-21	Spikevax	Feb-21	Spikevax	Nov-21	Spikevax	/	/	Dec-21
**17**	Vacc	female	51-60	Feb-21	Spikevax	Mar-21	Spikevax	Nov-21	Spikevax	/	/	Dec-21
**18**	Vacc	female	51-60	Jan-21	Spikevax	Feb-21	Spikevax	Nov-21	Spikevax	/	/	Dec-21
**19**	Vacc	male	61-70	Jan-21	Spikevax	Feb-21	Spikevax	Nov-21	Spikevax	/	/	Dec-21
**20**	Vacc	female	41-50	Feb-21	Spikevax	Mar-21	Spikevax	Nov-21	Spikevax	/	/	Dec-21
**21**	Vacc	female	61-70	Feb-21	Spikevax	Mar-21	Spikevax	Nov-21	Spikevax	/	/	Dec-21
**22**	Vacc	female	61-70	Jan-21	Spikevax	Feb-21	Spikevax	Nov-21	Spikevax	/	/	Dec-21
**23**	Vacc	female	21-30	Mar-21	Spikevax	Apr-21	Spikevax	Nov-21	Spikevax	/	/	Dec-21
**24**	Vacc	female	11-20	Mar-21	Spikevax	Apr-21	Spikevax	Nov-21	Spikevax	/	/	Dec-21
**25**	Vacc	male	61-70	Jan-21	Spikevax	Feb-21	Spikevax	Nov-21	Spikevax	/	/	Dec-21
**26**	Vacc	male	41-50	Jan-21	Spikevax	Feb-21	Spikevax	Nov-21	Spikevax	/	/	Feb-22
**27**	Vacc	female	41-50	Feb-21	Spikevax	Mar-21	Spikevax	Nov-21	Spikevax	/	/	Feb-22
**28**	Vacc	male	41-50	Feb-21	Spikevax	Mar-21	Spikevax	Dec-21	Spikevax	/	/	Mar-22
**29**	Vacc	female	31-40	Feb-21	Spikevax	Mar-21	Spikevax	Dec-21	Spikevax	/	/	Feb-22
**30**	Vacc	female	21-30	Feb-21	Spikevax	Mar-21	Spikevax	Nov-21	Comirnaty	/	/	Feb-22
**31**	Vacc	male	51-60	Feb-21	Spikevax	Mar-21	Spikevax	Dec-21	Comirnaty	/	/	Feb-22
**32**	Vacc	female	21-30	Feb-21	Spikevax	Mar-21	Spikevax	Nov-21	Comirnaty	/	/	Feb-22
**33**	Vacc	female	51-60	Feb-21	Spikevax	Mar-21	Spikevax	Nov-21	Spikevax	/	/	Feb-22
**34**	Vacc	male	31-40	Feb-21	Spikevax	Mar-21	Spikevax	Nov-21	Spikevax	/	/	Feb-22
**35**	Vacc	female	41-50	Feb-21	Spikevax	Mar-21	Spikevax	Dec-21	Spikevax	/	/	Feb-22
**36**	Vacc	female	41-50	Feb-21	Spikevax	Mar-21	Spikevax	Nov-21	Spikevax	/	/	Feb-22
**37**	Vacc	female	21-30	Jan-21	Spikevax	Feb-21	Spikevax	Nov-21	Comirnaty	/	/	Feb-22
**38**	Vacc	male	41-50	Jan-21	Spikevax	Feb-21	Spikevax	Nov-21	Spikevax	/	/	Feb-22
**39**	Vacc	female	51-60	Feb-21	Spikevax	Mar-21	Spikevax	Dec-21	Spikevax	/	/	Feb-22
**40**	Vacc	female	61-70	Feb-21	Spikevax	Mar-21	Spikevax	Dec-21	Spikevax	/	/	Feb-22
**41**	Vacc	female	61-70	Feb-21	Spikevax	Mar-21	Spikevax	Nov-21	Spikevax	/	/	Feb-22
**1**	O-Inf	female	31-40	/	/	/	/	/	/	Apr-22	BA.2	May-22
**2**	O-Inf	female	41-50	/	/	/	/	/	/	Apr-22	BA.2	May-22
**3**	O-Inf	male	21-30	/	/	/	/	/	/	Mar-22	BA.2	Jun-22
**4**	O-Inf	male	1-10	/	/	/	/	/	/	Apr-22	BA.2	Apr-22
**5**	O-Inf	female	21-30	/	/	/	/	/	/	Feb-22	BA.1	Feb-22
**6**	O-Inf	female	41-50	/	/	/	/	/	/	Jan-22	BA.1*	Aug-22
**7**	O-Inf	female	21-30	/	/	/	/	/	/	Mar-22	BA.2	Mar-22
**8**	O-Inf	female	51-60	/	/	/	/	/	/	Mar-22	BA.2	Sep-22
**9**	O-Inf	male	61-70	/	/	/	/	/	/	Aug-22	BA.5	Aug-22
**10**	O-Inf	male	21-30	/	/	/	/	/	/	Sep-22	BA.5	Oct-22
**11**	O-Inf	male	31-40	/	/	/	/	/	/	Mar-22	BA.2	Jul-22
**12**	O-Inf	male	81-90	/	/	/	/	/	/	Apr-22	BA.2	Jul-22
**13**	O-Inf	male	91-100	/	/	/	/	/	/	Aug-22	BA.5	Sep-22
**14**	O-Inf	female	31-40	/	/	/	/	/	/	Mar-22	BA.2	Jul-22
**15**	O-Inf	male	81-90	/	/	/	/	/	/	May-22	BA.2	Aug-22
**16**	O-Inf	female	81-90	/	/	/	/	/	/	Jun-22	BA.2/5	Aug-22
**17**	O-Inf	male	51-60	/	/	/	/	/	/	Aug-22	BA.5	Aug-22

**18**	O-Inf	female	31-40	/	/	/	/	/	/	Apr-22	BA.2	Jul-22
**19**	O-Inf	female	31-40	/	/	/	/	/	/	Jul-22	BA.5	Oct-22
**20**	O-Inf	female	21-30	/	/	/	/	/	/	Apr-22	BA.2	Jul-22
**21**	O-Inf	male	51-60	/	/	/	/	/	/	May-22	BA.2	Jul-22
**22**	O-Inf	female	61-70	/	/	/	/	/	/	Sep-22	BA.5	Oct-22
**23**	O-Inf	male	61-70	/	/	/	/	/	/	Mar-22	BA.2	Jul-22
**24**	O-Inf	male	61-70	/	/	/	/	/	/	Apr-22	BA.2	Jul-22
**25**	O-Inf	male	51-60	/	/	/	/	/	/	Aug-22	BA.5	Oct-22
**26**	O-Inf	female	51-60	/	/	/	/	/	/	Jun-22	BA.2/5	Sep-22
**27**	O-Inf	female	51-60	/	/	/	/	/	/	Aug-22	BA.5	Sep-22
**28**	O-Inf	male	51-60	/	/	/	/	/	/	Jan-22	BA.1*	May-22

Supplemental table 1) Demographic information with infection and vaccination time points. The table contains following information for each study participant (columns left to right in sequential order): pseudonym, group, age (as 10-year range), first dose vaccination date, first dose vaccine type, second dose vaccination date, second dose vaccine type, third dose vaccination date, third dose vaccine type, date of positive SARS-CoV-2 RT-PCR, the most prevalent omicron subvariant in Germany at the time of sampling (source data: https://ourworldindata.org/), date of sample collection. Sign "/" indicates that the information is not relevant for the individual. Asterisk* indicates that the omicron subvariant was determined by RNA sequencing.

Assay validation: competitive SARS-CoV-2 omicron RBD IgG ELISA

Reproducibility

	Sample 1	*Sample 2*	*Sample 3*	*Sample 4*	*Sample 5*
**Mean**	4,0	71,8	58,2	13,7	23,2
**SD**	0,4	11,8	9,8	1,7	3,0
**CV (%)**	10,3	16,5	16,8	12,3	13,1

Inter-assay precision: A total of 5 experiments each measuring 5 different plasma samples from previously uninfected and unvaccinated individuals infected with omicron SARS-CoV-2 were performed on different days to assess the inter-assay precision of the the competitive SARS-CoV-2 omicron RBD IgG ELISA. The output of this assay is the percentage of omicron but not wild-type RBD-binding IgG as a proportion of total omicron RBD-binding IgG. **The average coefficient of variation was 13,8%.**

Specificity

	**% of O not wild-type RBD-specific IgG**		**% of O not wild-type RBD-specific IgG**
Sample 1	0	*Sample 12*	0
Sample 2	0	*Sample 13*	0
Sample 3	0	*Sample 14*	0,6
Sample 4	0	*Sample 15*	0,8
Sample 5	1,1	*Sample 16*	0
Sample 6	0	*Sample 17*	0
Sample 7	1,1	*Sample 18*	0
Sample 8	0	*Sample 19*	0
Sample 9	0	*Sample 20*	0
Sample 10	0	*Sample 21*	0
Sample 11	0	*Sample 22*	0

22 plasma samples from wild-type-vaccinated individuals from before the emergence of omicron SARS-CoV-2 were analyzed with the competitive SARS-CoV-2 omicron RBD IgG ELISA. The output of this assay is the percentage of omicron but not wild-type RBD-binding IgG as a proportion of total omicron RBD-binding IgG. All selected samples were positive for omicron RBD IgG. **From 22 samples 18 were negative for omicron but not wild-type-specific IgG resulting in a specificity of 82%.** Positive readings are likely due to the presence of cross-reactive antibodies in the plasma of some individuals.

Sensitivity

	**% of O not wild-type RBD-specific IgG**
Sample 1	50,6
Sample 2	25,4
Sample 3	22,1
Sample 4	41,7
Sample 5	10,6
Sample 6	81,2
Sample 7	68,8
Sample 8	36,7
Sample 9	3,1
Sample 10	42,4
Sample 11	25,7
Sample 12	18,8
Sample 13	50,0
Sample 14	16,2
Sample 15	11,7

15 plasma samples from previously uninfected and unvaccinated individuals infected with omicron SARS-CoV-2 were analyzed with the competitive SARS-CoV-2 omicron RBD IgG ELISA. The output of this assay is the percentage of omicron but not wild type RBD-binding IgG as proportion of total omicron RBD-binding IgG. All analyzed samples were positive for omicron RBD IgG. **From 15 samples 15 were positive for omicron but not wild-type specific IgG resulting in sensitivity of 100%.**

Assay validation: competitive SARS-CoV-2 omicron plaque reduction neutralization assay

Reproducibility

	Sample 1	*Sample 2*	*Sample 3*	*Sample 4*	*Sample 5*
**Mean**	46,9	22,7	60,6	17,7	29,9
**SD**	9,4	6,1	17,9	4,3	5,7
**CV (%)**	20,0	26,9	29,5	24,5	19,1

Inter-assay precision: A total of 5 experiments each measuring 5 different plasma samples from previously uninfected and unvaccinated individuals infected with omicron SARS-CoV-2 were performed on different days to assess the inter-assay precision of the competitive SARS-CoV-2 omicron plaque reduction neutralization assay. The output of this assay is the percentage of omicron- but not wild-type-neutralizing antibodies as proportion of total omicron-neutralizing antibodies. **The average coefficient of variation was %.**

Specificity

	**% of O not wild-type RBD-specific IgG**		**% of O not wild-type RBD-specific IgG**
Sample 1	0	*Sample 12*	0
Sample 2	0	*Sample 13*	0
Sample 3	0	*Sample 14*	0
Sample 4	0	*Sample 15*	0
Sample 5	14,9	*Sample 16*	7,6
Sample 6	0	*Sample 17*	3,0
Sample 7	0,0	*Sample 18*	0
Sample 8	0	*Sample 19*	0
Sample 9	0	*Sample 20*	0
Sample 10	0	*Sample 21*	0
Sample 11	3,8	*Sample 22*	0

22 plasma samples from wild-type-vaccinated individuals from before the emergence of omicron SARS-CoV-2 were analyzed with the competitive SARS-CoV-2 omicron plaque reduction neutralization assay. The output of this assay is the percentage of omicron- but not wild-type-neutralizing antibodies as proportion of total omicron-neutralizing antibodies. All selected samples had detectable omicron-neutralizing antibodies. **From 22 samples 18 were negative for omicron- but not wild-type-neutralizing antibodies resulting in specificity of 82%.** Positive readings are likely due to the presence of cross-reactive antibodies in plasma of some individuals.

Sensitivity

	**% of O not wild-type RBD-specific IgG**
Sample 1	34,8
Sample 2	68,6
Sample 3	21,2
Sample 4	55,6
Sample 5	54,6
Sample 6	88,8
Sample 7	81,8
Sample 8	100,0
Sample 9	100,0
Sample 10	57,9
Sample 11	67,3
Sample 12	0,0
Sample 13	29,0
Sample 14	81,7
Sample 15	68,7
Sample 16	6,6
Sample 17	11,3
Sample 18	20,4

18 plasma samples from previously uninfected and unvaccinated individuals infected with omicron SARS-CoV-2 were analyzed with the competitive SARS-CoV-2 omicron plaque reduction neutralization assay. The output of this assay is the percentage of omicron- but not wild-type-neutralizing antibodies as a proportion of total omicron-neutralizing antibodies. All selected samples had detectable omicron-neutralizing antibodies. **From 18 samples 17 were positive for omicron- but not wild-type-neutralizing antibodies resulting in a sensitivity of 94%.**
