# Supplementary material for: Vaccination impairs de novo immune response to omicron breakthrough infection, a precondition for the original antigenic sin
Source: Nat Commun. 2024 Apr 10;15:3102. doi: 10.1038/s41467-024-47451-w (PMC11006949; doi:10.1038/s41467-024-47451-w)
Supplement: Supplementary file 3 — Reporting Summary [file 41467_2024_47451_MOESM3_ESM.pdf]

nature portfolio

Corresponding author(s): Jernej Pušnik

Last updated by author(s): 20.03.2024

Reporting Summary

Nature Portfolio wishes to improve the reproducibility of the work that we publish. This form provides structure for consistency and transparency in reporting. For further information on Nature Portfolio policies, see our Editorial Policies and the Editorial Policy Checklist.

Statistics

For all statistical analyses, confirm that the following items are present in the figure legend, table legend, main text, or Methods section.

n/a	Confirmed	
☐	☒	The exact sample size (*n*) for each experimental group/condition, given as a discrete number and unit of measurement
☐	☒	A statement on whether measurements were taken from distinct samples or whether the same sample was measured repeatedly
☐	☒	The statistical test(s) used AND whether they are one- or two-sided<br>*Only common tests should be described solely by name; describe more complex techniques in the Methods section.*
☐	☒	A description of all covariates tested
☐	☒	A description of any assumptions or corrections, such as tests of normality and adjustment for multiple comparisons
☐	☒	A full description of the statistical parameters including central tendency (e.g. means) or other basic estimates (e.g. regression coefficient) AND variation (e.g. standard deviation) or associated estimates of uncertainty (e.g. confidence intervals)
☐	☒	For null hypothesis testing, the test statistic (e.g. *F*, *t*, *r*) with confidence intervals, effect sizes, degrees of freedom and *P* value noted<br>*Give P values as exact values whenever suitable.*
☒	☐	For Bayesian analysis, information on the choice of priors and Markov chain Monte Carlo settings
☒	☐	For hierarchical and complex designs, identification of the appropriate level for tests and full reporting of outcomes
☒	☐	Estimates of effect sizes (e.g. Cohen's *d*, Pearson's *r*), indicating how they were calculated

Our web collection on statistics for biologists contains articles on many of the points above.

Software and code

Policy information about availability of computer code

Data collection	FACSDiva™ Software Version 8.0 (BD Bioscience)
Data analysis	GraphPad Prism software version 9.4.1. (681), FlowJo Software version 10.0.7 (TreeStar)

For manuscripts utilizing custom algorithms or software that are central to the research but not yet described in published literature, software must be made available to editors and reviewers. We strongly encourage code deposition in a community repository (e.g. GitHub). See the Nature Portfolio guidelines for submitting code & software for further information.

Data

Policy information about availability of data

All manuscripts must include a data availability statement. This statement should provide the following information, where applicable:

- Accession codes, unique identifiers, or web links for publicly available datasets
- A description of any restrictions on data availability
- For clinical datasets or third party data, please ensure that the statement adheres to our policy

The data contain information that could compromise the privacy of research participants. Data sharing restrictions imposed by national and transnational data protection laws prohibit the general sharing of data. However, upon submission of a proposal to the corresponding author; Jernej Pušnik (Jernej.Pusnik@ukbonn.de) and approval of this proposal by (i) the principal investigator, (ii) the Ethics Committee of the University of Bonn, and (iii) the data protection officer of the University Hospital Bonn, data collected for the study can be made available to other researchers. This process can take up to 8 weeks. The applicant will be instructed regarding the restrictions imposed on the use of the data. A source data file containing the statistics presented in the figures and a

Supplemental table containing demographic information are provided with this paper.

Research involving human participants, their data, or biological material

Policy information about studies with human participants or human data. See also policy information about sex, gender (identity/presentation), and sexual orientation and race, ethnicity and racism.

Reporting on sex and gender	Individuals were not discriminated by sex or gender when recruiting them for the study and allocating them to groups, however, we collected information about sex and gender. Moreover, the study was not designed to perform sex- or gender-based analyses.
Reporting on race, ethnicity, or other socially relevant groupings	We did not collect this infromation for the purpose of the study.
Population characteristics	The information we collected regarding population characteristics is available in supplemental table 1
Recruitment	A total of 106 individuals were included in this study. 87 were recruited by the occupational healthcare department of the University Hospital Bonn and 19 by the Emergency Medicine department of the University Göttingen in Germany. The first contact was established by telephone after which a written invitation and a consent form were sent to each participant. Individuals were divided into three groups according to their histories of exposure to SARS-CoV-2 antigens: individuals who had received three mRNA (encoding wild-type spike protein) vaccine doses and subsequently recovered from an omicron breakthrough infection (Vacc+O-Inf, n=37), individuals who received three mRNA (encoding wild-type spike protein) vaccine doses and were not infected with SARS-CoV-2 (Vacc, n=41), and individuals that did not get vaccinated but were infected with omicron (O-Inf, n=28). Age or sex was not among the selection criteria. Following gender distribution was observed between the groups: 65% females and 35% males for the Vacc+O-Inf group, 66% females and 34% males for the Vacc group, 50% females and 50% males for the O-Inf group and 57% females and 43% males for the subgroup of 7 O-Inf individuals with available PBMC samples. No significant differences in age distribution were observed between the groups (mean years±SD for O-Inf, Vacc+O-Inf, Vacc groups and a subgroup of 7 O-Inf individuals with available PBMC samples respectively: 50±21, 40 ±15, 47±14, 44±13). SARS-CoV-2 infections were confirmed by RT-PCR. During the time period of sample collection, the prevalence of omicron variants was >99% as assessed by sentinel seqeuencing. Detailed information on the vaccination, infection, and sampling time points as well as demographic information is provided in supplemental table 1. All individuals with omicron SARS-CoV-2 infection did not have previously confirmed SARS-CoV-2 infection. For the Vacc group only individuals without confirmed SARS-CoV-2 infection, and negative nucleocapsid ELISA results were included. Vaccinations of individuals included in this study were performed at the occupational healthcare department of the University Hospital Bonn. The cohort might be biased towards individuals with a higher degree of education and health awareness which is unlikely to significantly affect the results of this study.
Ethics oversight	The study was approved by the Ethics Committee of the Medical Faculty of the University of Bonn (ethics approval number 125/21) and the Ethics Committee of University Medical Center Goettingen (ethics approval number 21/06/22). All participants provided written informed consent. No compensation was provided for the participants.

Note that full information on the approval of the study protocol must also be provided in the manuscript.

Field-specific reporting

Please select the one below that is the best fit for your research. If you are not sure, read the appropriate sections before making your selection.

- [x] Life sciences
- [ ] Behavioural & social sciences
- [ ] Ecological, evolutionary & environmental sciences

For a reference copy of the document with all sections, see nature.com/documents/nr-reporting-summary-flat.pdf

Life sciences study design

All studies must disclose on these points even when the disclosure is negative.

Sample size	Sample sizes were determined by availability of the samples.
Data exclusions	No data were excluded
Replication	Standards and/or controls were measured during each experiment to ensure reproducibility of the method. All attempts at replication were successful. For ELISA experiments 8 dilutions of each sample were measured. Plasma neutralization assays were performed by measuring 12 dilutions of each sample. For flow cytometry experiments, no technical replicates were performed due to the scarcity of the samples.
Randomization	Participants were grouped based on their SARS-CoV-2 antigen exposure history
Blinding	Investigators received unique identifier codes (no personal names) for the samples, allocation to groups was done during the data analysis

Reporting for specific materials, systems and methods

We require information from authors about some types of materials, experimental systems and methods used in many studies. Here, indicate whether each material, system or method listed is relevant to your study. If you are not sure if a list item applies to your research, read the appropriate section before selecting a response.

Materials & experimental systems

n/a	Involved in the study
	☒ Antibodies
	☒ Eukaryotic cell lines
☒	Palaeontology and archaeology
☒	Animals and other organisms
	☒ Clinical data
☒	Dual use research of concern
☒	Plants

Methods

n/a	Involved in the study
☒	ChIP-seq
	☒ Flow cytometry
☒	MRI-based neuroimaging

Antibodies

Antibodies used	Human IgG Isotype Control, Invitrogen, 12-000-C, 100ng/ml, HRP-conjugated anti-IgG antibody (Goat anti-Human IgG (Heavy chain) Secondary Antibody, HRP, Invitrogen, A18805) diluted 1:8000; BD FastImmune™ CD28/CD49d, BD, 347690, 1 µg/ml); anti-CD3-APC-Cy7 (clone UCHT1; Biolegend, 300426, diluted 1:40), anti-CD4-BV786 (clone SK3; BD Bioscience, 344642, diluted 1:40), anti-IFNγ-PE (clone B27; Biolegend, 506507, diluted 1:40), anti-TNFα-BV421 (clone Mab11; Biolegend, 502932, diluted 1:80); anti-CD8-PE-Cy7 (clone SK1, Biolegend, 344712, diluted 1:80); anti-IgG-BV421 antibody (clone G18-145, Biolegend, 562581, diluted 1:20); antibodies blocking human Fc receptors (FcR block, Miltenyi Biotec, 130-059-901, diluted 1:10); anti-CD3-BV510 (clone UCHT1, Biolegend, 300448, diluted 1:40), anti-CD27-BV605 (clone O323, Biolegend, 302830, diluted 1:20), anti-IgM-BV785 (clone MHM-88, Biolegend, 314544, diluted 1:20), anti-IgA-VioBright 515 (clone REA1014, Miltenyi Biotec, 130-116-886, diluted 1:40), anti-CD21-PE-Cy7 (clone Bu32, Biolegend, 354912, diluted 1:160), and anti-CD19-APC-Cy7 (clone HIB19, Biolegend, 302218, diluted 1:80).
Validation	For flow cytometry experiments antibodies were titrated in following manner: For every fluorescently-labeled antibody eight 2-fold dilutions starting from 1:10 were prepared and incubated with PBMC of healthy donors. The samples were stained 15min at 4°C, washed with PBS and acquired on flow cytometer. To find the optimal concentration staining index was calculated as the ratio of the separation between the positive population and the negative population (difference in MFIs), divided by two times the standard deviation of the negative population. For ELISA experiments secondary antibodies conjugated to HRP were tested as follows: The overall ELISA procedure was performed as described in the manuscript. Seven dilutions of a plasma sample confirmed seropositive for anti-SARS-CoV-2 IgG were prepared and applied to the rows of a pre-coated 96-well ELISA plate. Subsequently, eleven 2-fold dilutions of secondary HRP-conjugated antibodies starting with 1:250 and a negative control without the antibody were added to the plate columns. The ELISA was than developed as described. The highest secondary antibody dilution that gave maximal signal increase over the background (no sample control) for most of the sample dilutions was used in the further assays. Validation of antibodies was performed by the manufacturer, and is provided on their websites: https://www.biolegend.com/, https://www.bd.com/en-us, https://www.thermofisher.com, https://www.miltenyibiotec.com/.

Eukaryotic cell lines

Policy information about cell lines and Sex and Gender in Research

Cell line source(s)	Vero E6; ATCC
Authentication	Cell line was not authenticated
Mycoplasma contamination	Cell line was negative for mycoplasma
Commonly misidentified lines (See ICLAC register)	No commonly misidentified cell line was used for the study.

Clinical data

Policy information about clinical studies

All manuscripts should comply with the ICMJEguidelines for publication of clinical research and a completedCONSORT checklist must be included with all submissions.

Clinical trial registration	Study does not involve clinical trials/clinical trial associated data.
Study protocol	Study does not involve clinical trials/clinical trial associated data.
Data collection	Study does not involve clinical trials/clinical trial associated data.
Outcomes	Study does not involve clinical trials/clinical trial associated data.

Plants

Seed stocks: Study does not involve plants.

Novel plant genotypes: Study does not involve plants.

Authentication: Study does not involve plants.

Flow Cytometry

Plots

Confirm that:

- [x] The axis labels state the marker and fluorochrome used (e.g. CD4-FITC).
- [x] The axis scales are clearly visible. Include numbers along axes only for bottom left plot of group (a 'group' is an analysis of identical markers).
- [x] All plots are contour plots with outliers or pseudocolor plots.
- [x] A numerical value for number of cells or percentage (with statistics) is provided.

Methodology

Sample preparation	Human PBMCs were used as samples, for processing details please look at the Methods section of the manuscript
Instrument	BD FACS Celesta, IC.nr:94915
Software	FACSDiva™ Software Version 8.0 (BD Bioscience), FlowJo Software version 10.0.7 (TreeStar)
Cell population abundance	No sorting was performed
Gating strategy	Gating strategy is provided as supplemental material.

- [x] Tick this box to confirm that a figure exemplifying the gating strategy is provided in the Supplementary Information.
